# Prognostic Modeling of Tricuspid Valve Regurgitation Outcomes Using Machine Learning-Based Survival Analysis

**DOI:** 10.3390/jcm15103859

**Published:** 2026-05-17

**Authors:** Sepehr Janghorbani, Pablo Villar Calle, Prianca Tawde, Jonathan W. Weinsaft, Jiwon Kim, Bobak Mosadegh

**Affiliations:** 1 Dalio Institute of Cardiovascular Imaging, Weill Cornell Medicine, New York, NY 10021, USA; 2 Department of Radiology, Weill Cornell Medicine, New York, NY 10021, USA; 3 Division of Cardiology, Weill Cornell Medicine, New York, NY 10021, USA; 4 New York Presbyterian Hospital, New York, NY 10021, USA

**Keywords:** tricuspid regurgitation, survival analysis, valvular disease, machine learning, deep learning

## Abstract

**Background:** Tricuspid regurgitation (TR) is a common valvular heart condition associated with significantly increased mortality. It is often underdiagnosed and undertreated due to limited insight into patient-specific risk prediction and optimal timing of intervention. Machine learning (ML) methods offer the potential to address these gaps by identifying high-risk patients, estimating survival probabilities, and uncovering key risk markers that influence outcomes. **Methods:** We developed and evaluated models to predict survival curves for a cohort of 949 patients with moderate or severe TR. Three modeling approaches were compared: Cox proportional hazards (Cox PH), Random Survival Forests (RSF), and DeepSurv (a deep learning-based survival model). Models were trained on clinical and imaging features extracted from cardiac magnetic resonance (CMR) studies and patient records. Performance was assessed using the concordance index (C-index) and time-dependent area under the receiver operating characteristic curve (AUC). Kaplan–Meier analysis and multivariable Cox regression were used to identify significant predictors of mortality. **Results:** RSF achieved the best predictive performance with a C-index of 78% and AUC of 82%, followed by DeepSurv (C-index 72%, AUC 78%) and Cox PH (C-index 66%, AUC 76%). Predicted survival curves for low- and high-risk groups demonstrated clear separation, underscoring the models’ ability to distinguish patient risk. Key predictors of poor survival included older age, tobacco exposure, right ventricular dilation and hypertrophy, right atrial enlargement, and the presence of non-ischemic myocardial fibrosis. These features were independently associated with elevated mortality risk and showed distinct survival differences in Kaplan–Meier analysis. **Conclusions:** Machine learning-based survival models, particularly RSF and DeepSurv, offer beneficial tools for individualized risk stratification in patients with advanced TR. Structural abnormalities of the right heart and myocardial fibrosis were among the most significant predictors of mortality, highlighting the importance of early detection and timely intervention. Integrating AI-driven survival prediction into clinical workflows could potentially benefit decision-making and enable more personalized management of TR.

## 1. Introduction

Tricuspid regurgitation (TR) is one of the most prevalent valvular heart conditions worldwide [1]. While mild TR is relatively common—affecting approximately 65–85% of the general population—and often clinically silent, moderate-to-severe TR is associated with substantially worse outcomes. Studies have demonstrated a two- to three-fold increase in mortality over 1 to 4 years among patients with significant TR. Moreover, TR contributes to worsening heart failure symptoms and elevates the risk of hospitalization [2,3]. Despite its clinical significance, TR has historically been underdiagnosed and undertreated, leading to delayed interventions and poorer prognoses [4]. Early detection and timely management are therefore critical for improving outcomes and maintaining quality of life. Diagnosis of TR typically involves a combination of clinical assessment and imaging techniques. Transthoracic echocardiography (TTE) and transesophageal echocardiography (TEE) are commonly used for initial evaluation of valve structure and function [5]. In recent years, cardiac magnetic resonance imaging (CMR) has emerged as another valuable diagnostic modality, offering detailed visualization of the tricuspid valve and right ventricular anatomy. CMR is a powerful diagnostic tool since it provides precise quantification of regurgitant volume, assessment of right ventricular function, and detection of myocardial fibrosis [6]. Despite recent advancements, one of the major challenges in the management of tricuspid regurgitation (TR) is determining the optimal timing for intervention. Delaying treatment can lead to irreversible right ventricular dysfunction and higher mortality, whereas intervening too early may expose patients to unnecessary procedural risks [7,8]. In this context, artificial intelligence (AI) presents a promising opportunity to enhance clinical decision-making. Current assessments largely depend on echocardiographic parameters and clinician judgment, which can vary significantly due to the subjective nature of interpretation and variability in guidelines. AI, by contrast, has the potential to identify novel prognostic markers, predict survival outcomes, and stratify patients based on their likelihood of benefiting from earlier intervention. This approach enables more personalized and timely treatment strategies, ultimately aiming to improve outcomes in patients with TR.

Machine learning (ML) has demonstrated substantial utility across a wide range of applications in both the diagnosis and prediction of cardiovascular diseases. For instance, ML models such as Random Forest and Support Vector Machines (SVMs) have been effectively used to predict chronic heart disease [9,10,11]. ML has also been successfully applied in the prediction of atrial fibrillation [12], early diagnosis and prognosis of heart failure [13,14], and the assessment of coronary artery disease [15,16]. In one study, researchers employed eight different ML models to predict both 3-month readmission and 6-month mortality rates in heart failure patients [14]. Another study applied ML to predict heart failure in patients with premature myocardial infarction and developed a practical risk-scoring system for early identification and intervention [17]. ML has also been applied to vascular conditions, such as predictive modeling and risk analysis for peripheral vascular disease [18]. Additionally, using ML on electrocardiogram (ECG) data has shown potential in detecting structural heart disease up to one to two years earlier than traditional diagnostic methods [19]. Beyond diagnosis and prediction, AI has also facilitated the discovery of novel disease markers—for example, the identification of new mortality markers in patients with acute coronary syndrome (ACS) [20]. The application of AI in heart valve disease has also been explored, though most existing studies focus primarily on mitral valve disorders. Furthermore, these studies often model clinical outcomes as binary classification problems (e.g., mortality vs. survival), rather than using survival analysis. However, survival modeling provides a more comprehensive view by modeling the entire survival curve over time [21,22,23,24].

Beyond diagnostic classification, AI is increasingly being explored across procedural cardiology, including image interpretation, risk stratification, procedural planning, intraprocedural support, and postprocedural outcome prediction [25]. For example, in electrophysiology, AI can help localize arrhythmogenic substrate, guide AF ablation targets, reduce fluoroscopy/procedure time, and predict recurrence after catheter ablation [26,27,28]. In the catheterization laboratory, AI-assisted angiographic analysis can support FFR estimation, PCI planning, stent optimization, and selection between percutaneous and surgical strategies [26,29]. In patients with advanced TR, similar approaches could help integrate imaging, clinical, and outcome data to guide earlier referral, Heart Team discussion, and selection of candidates for transcatheter intervention.

In this study, we present machine learning-based models for survival prediction of patients with moderate-to-severe tricuspid regurgitation. We run our survival analysis on a previously curated cohort of 949 patients with advanced (≥moderate) tricuspid regurgitation (TR), with 144 clinical and imaging-derived features. Our analysis includes both linear models, such as the Cox proportional hazards model [30] as well as more complex nonlinear approaches, including Random Survival Forests [31] and deep learning methods based on DeepSurv [32]. Using these models, we have identified key features contributing to the predictive model and our best model was able to achieve a time-dependent AUC of 82% and concordance index of 78%.

## 2. Materials and Methods

### 2.1. Dataset Curation

The study cohort comprised 949 patients with advanced (≥moderate) functional tricuspid regurgitation (TR) who underwent clinical cardiovascular magnetic resonance (CMR) imaging at NewYork-Presbyterian/Weill Cornell Medical Center between July 2005 and June 2024. The study was approved by the institutional review board, with a waiver of informed consent granted due to the retrospective study design. Patients with congenital heart disease, prior tricuspid valve surgery, or primary tricuspid valve pathology (including prolapse, rheumatic involvement, or leaflet perforation) were excluded. Demographic characteristics, clinical variables, medical history, and medication use were obtained from the electronic medical record. All patients underwent a standardized clinical CMR protocol using steady-state free-precession cine imaging (typical parameters: repetition time, 3.5 ms; echo time, 1.6 ms; flip angle, 60°; in-plane spatial resolution, 1.9 × 1.4 mm). Following cine imaging, gadolinium contrast (0.15–0.20 mmol/kg) was administered intravenously. Phase-contrast CMR was performed approximately 10 min after contrast administration using an inversion-recovery sequence. Cine and late gadolinium enhancement (LGE) images were acquired in matching short- and long-axis planes. Short-axis cine images were obtained from the mitral valve annulus to the left ventricular apex using contiguous slices (typical slice thickness, 6 mm; interslice gap, 4 mm). Standard long-axis images were acquired in the 2-, 3-, and 4-chamber orientations. Quantification of tricuspid regurgitation, TR, severity was assessed by experienced CMR readers using regurgitant volume (>30 mL) and/or regurgitant fraction (>16%). Regurgitant volume was determined according to guideline-recommended methods: by differential forward stroke volume and right ventricular (RV) stroke volume when phase-contrast flow data of the pulmonic (or aortic) valve were available; or by differential RV and left ventricular (LV) stroke volume in the absence of significant mitral or aortic regurgitation. Late gadolinium enhancement, LGE, was evaluated using the standard 17-segment model. For each segment, LGE burden was graded (0 = no hyperenhancement; 1 = 1–25%; 2 = 26–50%; 3 = 51–75%; 4 = 76–100%). Global LGE burden was recorded as the sum of involved segments. LGE pattern was categorized as ischemic (subendocardial or transmural) or non-ischemic (midmyocardial or epicardial). Non-ischemic LGE was defined as hyperenhancement involving the midmyocardial and/or subepicardial layers, particularly within the interventricular septum. Data collection and analysis followed procedures consistent with previously published methodologies. More details on data collection can be found here [33].

All-cause mortality was determined through queries of the Social Security Death Index and institutional records. Follow-up time was calculated from the date of CMR to the date of death or last documented clinical contact. The baseline characteristics of the overall cohort are presented in Table 1.

The full study cohort consists of 949 patients. The mean age of the patients was 63.7 ± 15.8 years with a balanced gender distribution (51.3% male). The cohort had a high prevalence of cardiovascular risk factors including hypertension (64.3%), hyperlipidemia (52.2%), atrial fibrillation (39.4%) and coronary artery disease (47.3%). In total, 37.5% of the population also had some prior cardiac intervention and 16.3% had some prior valve intervention.

Based on the cardiac imaging parameters, the cohort demonstrates notable cardiac structural and functional abnormalities. The average left ventricular end-diastolic volume (LV EDVi) is elevated at 85.8 ± 37.5 mL/m^2^, with a correspondingly high end-systolic volume (LV ESVi) of 47.8 ± 36.9 mL/m^2^, suggesting impaired systolic function. Furthermore, the average right ventricular end-diastolic volume (RV EDVi) is elevated at 104.9 ± 38.5 mL/m^2^, with a correspondingly high end-systolic volume (RV ESVi) of 59.6 ± 31.7 mL/m^2^, suggesting impaired systolic function.

This is further supported by a reduced left ventricular ejection fraction (LV EF) of 49.6 ± 18.6% and right ventricular ejection fraction (RV EF) of 45.3 ± 12.8%. Right ventricular (RV) dilation was observed in 44.4% of patients, while RV hypertrophy was present in 7.7%, indicating strain on the right heart. Left ventricular hypertrophy and dilation are seen in 33.3% and 25.0% of the cohort, respectively. Notably, atrial enlargement was common, with left atrial (LA) and right atrial (RA) enlargement present in 67.2% and 84.8% of patients, respectively. Notably, nearly one-fourth of patients demonstrated presence of ischemic scar (26.4%), non-ischemic fibrosis (NIF 27.6%) and non-ischemic septal fibrosis (NIsF 26.1%). Table S1 in the Supplementary Materials shows the percentage of missing values for each variable. To be consistent with best practice, we used median-value imputation for numerical variables and missing-category imputation for categorical variables.

**Figure 1:**
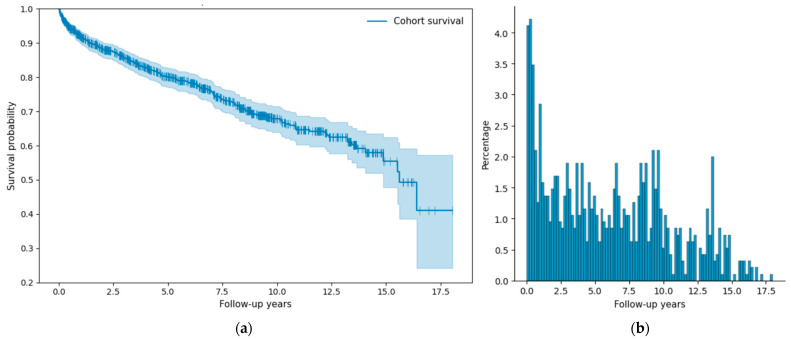
(**a**): Cohort censoring distribution. (**b**): Distribution of follow-up times of the patient cohort.

Out of 949 patients, 242 were dead (194 in the training cohort (25.3%) and 48 in the held-out cohort (26.2%)), and out of the remaining 707, 423 were alive at the end of study period (343 in the training cohort (44.8%) and 80 in the held-out cohort (43.7%)) and 284 were lost to follow-up (229 in the training cohort (29.9%) and 55 in the held-out cohort (30.1%)). The median follow-up time for the cohort was (5.53) with an IQR of 7.34, whereas the median potential follow-up estimated using the reverse Kaplan–Meier method was 7.76 years. Figure 1a shows the censoring distribution. In order to minimize the bias in the study, patients without a documented death were censored at the last date they were known to be alive or, if still under observation, at the administrative end of the study period. Figure 1b further illustrates the distribution and completeness of follow-up data in the survival analysis. The distribution of follow-up times is notably skewed, with many of the patients having relatively short follow-up durations—most under two years—and a sharp decline in frequency beyond that point. Such characteristics highlight the importance of employing time-to-event prediction models instead of simple binary classifiers. The skewed nature of the follow-up data presents challenges for conventional binary classification approaches, which only predict whether an event will occur, without accounting for the timing of the event. In contrast, survival modeling is better suited to this context, as it explicitly models the time until an event occurs, thereby providing more detailed and clinically actionable insights. It is also necessary to mention that as in most survival analyses, the number of patients remaining at risk decreases over time; consequently, estimates at later time points are based on fewer observations and may be noisier and less precise. Therefore, longer-horizon performance estimates should be interpreted with appropriate caution, particularly at time points where the number of patients at risk is small.

### 2.2. Preprocessing and Feature Selection

**Figure 2:**
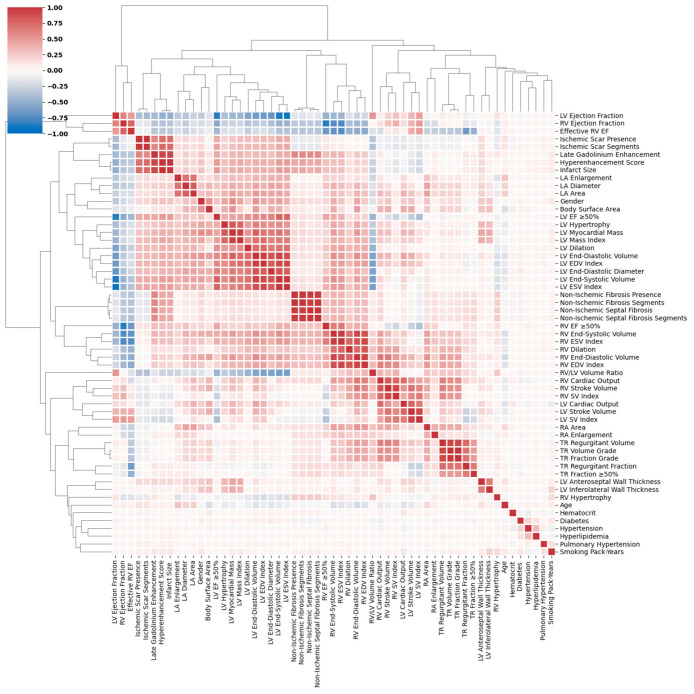
Correlation heatmap of extracted features. Red represents positive correlation while blue indicates negative correlation. Clinically or functionally related groups of variables are grouped together using hierarchical clustering.

As the first step in preprocessing, we identified and removed 57 of the features with minimal informational value, resulting in a reduced set of 87 features. Specifically, features with very low variance or entropy, having over 90% identical entries, were removed, as they provide limited discriminative power for modeling. We also noticed that some of the extracted features showed high feature redundancy. More specifically, inter-feature correlation was computed and features with a Spearman correlation coefficient >0.85 were also excluded, resulting in a set of 65 features. Figure 2 illustrates the correlation heatmaps across features. For better clarity, features with higher correlation are grouped together using hierarchical clustering to help reveal clinically or functionally related groups of variables. These correlations are either due to redundant representations of the same underlying variable (e.g., continuous vs. binary encoding) or from shared dependence on latent physiological or pathological processes.

For instance, the tricuspid regurgitation (TR) fraction shows an inverse relationship with right ventricular ejection fraction (RVEF), since an increase in TR fraction means a larger proportion of the RV stroke volume is lost backward into the right atrium, leading to a decline in effective RVEF. For the remaining missing values, we used a hybrid imputation strategy by replacing the continuous features with the median and an extra missing category for categorical features. In the final step of our feature selection pipeline, we employed the backward elimination feature selection technique, a wrapper-based feature selection method, on an initial set of clinically relevant features to further refine the feature set to be most informative. This iterative process allowed us to identify a final feature set (Provided in Supplementary Material) that demonstrated the greatest predictive value for downstream tasks, while minimizing redundancy and overfitting risk.

### 2.3. Statistical and Machine Learning Methods

Although survival prediction can be framed as a binary classification problem, this approach oversimplifies the continuous nature of time-to-event outcomes. Binary classification ignores the ongoing risk associated with each time point and fails to incorporate the varying lengths of patient follow-up. Additionally, choosing a fixed time threshold introduces subjectivity, and data from patients who are censored before that time becomes uninformative in a binary framework. In contrast, survival models are able to appropriately handle censored data and make use of all available follow-up information, allowing for more robust and interpretable risk estimation. In this study, we explored three families of survival models:

o Cox proportional hazards model (linear);
o Random Survival Forest Model (nonlinear/tree-based);
o Deep learning-based survival model (nonlinear/Neural Net-based).

#### 2.3.1. Cox PH Model

The Cox proportional hazards (Cox PH) model is one of the most widely used methods for survival analysis, particularly due to its ability to model the effect of covariates without requiring a specific form for the baseline hazard function. The model expresses the hazard function *h(t∣X)* as the product of a baseline hazard *h*_0_*(t)* and a linear combination of covariates formulated as

$$h(t|X)=h_{0}(t)\cdot \exp\left(\beta_{1}X_{1}+\beta_{2}X_{2}+\ldots +\beta_{p}X_{p}\right)$$

where *h*_0_*(t)* is the baseline hazard function and *β_i_* are the coefficients which are estimated using a partial likelihood objective.

#### 2.3.2. Random Survival Forestss

Although the Cox PH model is widely used in survival analysis, it only assumes a linear relationship between covariates and the log hazard function. However, for datasets with more complex and high-dimensional features, these assumptions could be limiting. Random Survival Forests (RSF) is a nonparametric method that makes no such assumptions, allowing nonlinear effects and higher-order interactions. Furthermore, they are more robust to the presence of multicollinearity and missing data and naturally accommodate clinical data, providing individual survival curves rather than relative risk scores. Because of this, these algorithms can capture higher-order nonlinear relationships among the features as well. RSF estimates the survival curve by constructing multiple decision trees using bootstrapping to find the best splits that maximize the survival differences between nodes. The following equation illustrates the ensemble nature of this algorithm, where each tree’s survival is computed by feeding the sample into B trees and averaging the output:

$${\hat{H}}_{\mathrm{RSF}}(t|X)=\frac{1}{B}\overset{B}{\underset{b=1}{\sum }}{\hat{H}}_{b}(t|X)\;,\;{\hat{S}}_{\mathrm{RSF}}(t|X)=\exp\left(-{\hat{H}}_{\mathrm{RSF}}(t|X)\right)$$

The hyperparameters of the Random Survival Forests were selected to enhance predictive stability while minimizing overfitting. The model was trained using 300 trees to improve robustness. To ensure that both node splits and terminal nodes were supported by sufficient patient counts, the minimum number of samples required for a split was set to 15. Furthermore, tree complexity was constrained by setting max depth to 6 and max_leaf_nodes to 600, which helped limit overfitting.

#### 2.3.3. Deep Learning-Based Survival Model (DeepSurv)

DeepSurv is a deep learning-based extension of the Cox proportional hazards (PH) model, which replaces the model’s linear covariate assumption with a neural network capable of learning complex, nonlinear interactions among input features. This added flexibility enables DeepSurv to capture intricate relationships within high-dimensional clinical and imaging data that may not be adequately modeled using traditional Cox regression. As a result, it is particularly well-suited for heterogeneous patient populations, where risk factors may interact in nonlinear or hierarchical ways to influence survival outcomes. The input to this model is patient features (e.g., age, lab values, etc) and uses a neural network-based model to estimate the predicted risk score (log-hazard score) for each patient, which is used to estimate survival differences and support personalized treatment decisions.

Model performance was evaluated using the concordance index (C-index) and time-dependent area under the receiver operating characteristic curve at specific time points. Kaplan–Meier survival curves were generated to evaluate survival differences between subgroups. To evaluate the models, we split the dataset into an 80–20% train–test split, reserving 20% of the data as held-out test set. The hyperparameters were optimized using 10-fold cross-validation.

During the preparation of this manuscript, the authors used ChatGPT 5.4 solely for editing, grammar correction, and improving the clarity of the English language. All content was originally written by the authors. The authors reviewed and edited all AI-assisted suggestions and take full responsibility for the content of this publication.

## 3. Results

Table 2 contains the predictive performance of different models. Among the evaluated models, Random Survival Forests (RSF) demonstrated the highest predictive performance, achieving a concordance index of 78% and a time-dependent Area Under the Curve (AUC) of 82%. DeepSurv had a concordance index of 72% and an AUC of 78%, and the traditional Cox proportional hazards model yielded a concordance of 66% and an AUC of 76%. These results suggest that nonlinear and ensemble-based approaches, such as RSF and DeepSurv, have the potential to provide slightly better discrimination and risk stratification in survival prediction. This is expected since DeepSurv and Random Survival Forests are able to model nonlinear interactions as opposed Cox PH model, which only models linear components.

**Figure 3:**
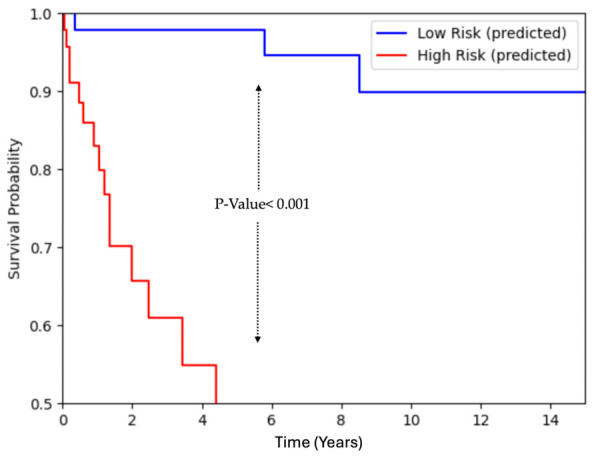
Survival curves for high-risk (red) and low-risk (blue) patients using the RSF predictive model.

Furthermore, to qualitatively assess Random Forest’s predictive performance, we examined its ability to estimate survival functions on the held-out test set. Patients were categorized into low-risk and high-risk groups based on the model’s survival prediction—specifically, those within the top quartile of risk scores were labeled as high-risk, and those within the bottom quartile of risk scores were labeled as low-risk. After stratifying the patients into high- vs. low-risk groups based on the model’s prediction, we plotted the empirical KM curves to observe the actual plots of patient survival (shown in Figure 3). The results demonstrate a clear separation between the two risk groups: the low-risk group (blue curve) maintains a consistently higher survival probability over time, while the high-risk group (red curve) shows a steeper decline. This separation highlights the model’s predictive ability to distinguish between patients with different risk factors, highlighting its potential clinical utility.

Another important aspect of survival analysis is identifying causal factors contributing to patient survival. Table 3 includes the coefficients of different covariates estimated by the Cox PH model. These covariates can be used to further interpret and explain the clinical factors affecting survival.

## 4. Discussion

The clinical literature consistently demonstrates that increasing severity of tricuspid regurgitation (TR) is associated with worse clinical outcomes, providing a strong rationale for developing dedicated mortality prediction models in patients with clinically significant TR [34,35]. Prior studies have established the prognostic importance of TR across diverse patient populations. For example, Kelly et al. showed that preoperative or intraoperative TR severity in cardiac surgery patients was independently associated with long-term mortality, highlighting the clinical relevance of TR grading [36]. Similarly, Messika-Zeitoun et al., using a large electronic health record dataset of patients with heart failure, identified that the presence of TR was associated with increased mortality risk [37]. Further supporting these findings, Benfari et al. demonstrated that in patients with heart failure with reduced ejection fraction (HFrEF), increasing severity of functional TR was independently associated with higher long-term mortality compared with trivial TR [38]. While this study reinforces mortality as a meaningful endpoint, its focus was limited to functional TR within HFrEF rather than a broader population with moderate-to-severe TR. In addition, Fender et al. evaluated patients with isolated TR and found that this group experiences excess mortality and frequent heart failure hospitalizations; however, their work was primarily observational and did not aim to develop predictive models [39]. Attempts have also been made to create risk stratification tools for patients with TR. For instance, Lara-Breitinger et al. introduced the TRIO score as a simple clinical risk assessment tool for patients with significant TR [40]. While useful for bedside stratification, such approaches rely on simplified scoring systems and may not capture complex nonlinear relationships between variables. In contrast, our approach leverages proper survival modeling techniques to better account for these complexities and improve individualized risk prediction.

By contrast, much of the work that uses more advanced models such as deep learning-based architectures for tricuspid regurgitation (TR) has focused on detection, grading, and phenotyping rather than mortality prediction, underscoring the distinct contribution of our study. For example, the DELINEATE-Regurgitation study developed an artificial intelligence system to evaluate aortic, mitral, and tricuspid regurgitation and to stratify the risk of mitral regurgitation (MR) progression. Although TR was included in the assessment, the study’s primary prognostic objective was centered on MR progression rather than mortality prediction in patients with moderate-to-severe TR [41]. Similarly, Vrudhula et al. proposed an automated deep learning workflow capable of identifying apical four-chamber color Doppler videos and detecting clinically significant TR from full transthoracic echocardiography studies, demonstrating strong performance across temporally and geographically distinct cohorts; however, the endpoint was TR detection and severity classification rather than survival prediction [42]. Several electrocardiogram (ECG)-based deep learning models have also been developed. SPEED-TR introduced a transformer-based model for detecting TR from standard 12-lead ECGs using multicenter datasets, and Chang et al. proposed a similar ECG-based approach for identifying significant TR. However, these models are designed for screening and detection, not for predicting mortality in patients with established moderate-to-severe TR [43]. Likewise, Xie et al. developed an end-to-end deep learning system using continuous-wave Doppler spectra to assess TR severity, but its objective was severity classification rather than detailed prognostic survival modeling [44]. In parallel, imaging-based approaches such as TRI-PLAN, automated CT annulus extraction, and CT-derived anatomic predictors have advanced right-heart assessment and transcatheter procedural planning. While highly relevant to TR imaging, these studies focus on anatomical characterization, procedural guidance, or procedural outcomes rather than pre-treatment mortality prediction [45,46,47]. Similarly, comparative repair studies, transcatheter valve replacement trials, and investigations of isolated tricuspid surgery have provided important insights into treatment outcomes and prognostic markers—such as right ventricular strain—but these remain downstream of the central question addressed in our study [48,49,50].

The body of work most closely related to our study is the literature on predictive modeling in moderate-to-severe tricuspid regurgitation (TR). For example, Badano et al. used advanced echocardiography and cluster analysis to identify three phenogroups of secondary TR with distinct risk profiles, highlighting the heterogeneity and prognostic structure of this condition [51]. However, unsupervised phenogrouping is conceptually different from our objective, which is individualized time-to-event mortality prediction. In this context, survival forests are more directly predictive, clinically actionable, and better suited to inform intervention because they estimate patient-specific risk over time. More broadly, most prior TR risk stratification studies have relied on conventional machine learning methods, less comprehensive feature sets, and simplified survival models. Heitzinger et al. developed a streamlined machine learning approach for risk stratification in heart failure patients with secondary TR, making their work methodologically relevant to ours [52]. However, their study was limited to heart failure patients with secondary TR and was designed as a simplified clinical stratification tool rather than a deep survival framework applied to a broader population with moderate-to-severe TR. Other related studies have focused primarily on intervention-specific cohorts. Stocker et al. examined patients undergoing transcatheter tricuspid valve edge-to-edge repair (TTVR) and aimed to identify hemodynamic predictors of postprocedural mortality [53]. Compared with our study, their cohort was substantially smaller (236 patients), limited to patients already selected for TTVR, and analyzed using conventional linear models such as Cox regression. In addition, their study did not incorporate CMR-derived features. Similarly, Fortmeier et al. used survival modeling for outcome prediction in patients undergoing transcatheter tricuspid intervention, and Brener et al. investigated noninvasive estimation of right ventricular–pulmonary arterial coupling in patients being considered for transcatheter tricuspid intervention. Taken together, these studies are centered on procedural referral or intervention cohorts rather than on general mortality prediction in patients with moderate-to-severe TR irrespective of treatment status. Moreover, unlike our study, they did not include CMR features, which may provide important prognostic information, including the presence of ischemic and non-ischemic myocardial scar [54,55]. The closest prior work to our study is that of Deb et al., who investigated a retrospective cohort of patients with at least moderate tricuspid regurgitation identified on echocardiography, with the goal of predicting long-term all-cause mortality. Their study aimed to determine whether machine learning methods could improve risk stratification and survival prediction compared with more conventional survival-analysis approaches in patients with significant TR. The authors evaluated several survival-prediction models, including traditional Cox-based methods and tree-based machine learning approaches, and reported a best C-statistic of 0.75 for mortality prediction. This performance was slightly lower than the C-index of 0.78 observed in our study, which may be explained by the inclusion of CMR-derived variables in our model, particularly ischemic scar and non-ischemic septal fibrosis, that may provide additional prognostic information beyond standard clinical and echocardiographic features [56].

Table 2 reports the predictive performance of the models, demonstrating that nonlinear approaches such as Random Survival Forests (RSF) and DeepSurv tend to outperform the traditional Cox proportional hazards model. This likely reflects the complex and interrelated nature of factors influencing mortality in patients with moderate-to-severe tricuspid regurgitation. Although all models use the same set of input features, RSF and DeepSurv can show higher predictive power. This is primarily because, unlike the Cox PH model—which represents risk using only linear additive effects—these models can capture more complex, higher-order interactions. This is particularly important when the true underlying risk surface is nonlinear. RSF reduces the bias of linear models by learning flexible, nonparametric splits that automatically model nonlinearities and interactions. Its ensemble structure further stabilizes predictions and improves risk ranking in the presence of noisy or high-dimensional covariates. DeepSurv similarly replaces the linear predictor with a neural network, allowing the model to learn intricate feature compositions and interaction structures, directly optimizing a risk score for survival ranking. When true hazard relationships deviate from linear additivity, both RSF and DeepSurv can generate better-calibrated risk orderings, resulting in higher concordance compared to the linear Cox PH baseline. Overall, RSF has stronger predictive accuracy and robustness to complex data structures, although it is limited by lower interpretability and a potential risk of overfitting if not carefully tuned. Similarly, Deepsurv has relatively high flexibility and the ability to learn complex latent risk patterns, while its drawbacks include higher data and tuning requirements, reduced interpretability, and sensitivity to sample size and hyperparameter selection. In contrast, the Cox proportional hazards model showed lower predictive performance, likely due to its assumption of linear covariate effects and proportional hazards over time; however, it remains advantageous for its interpretability and clinical familiarity. Overall, the differences in performance across the three methods highlight a tradeoff between flexibility and interpretability, with RSF benefiting most from the heterogeneous and nonlinear characteristics of this patient population, DeepSurv offering a balance between complexity and performance, and Cox providing greater transparency but reduced predictive accuracy.

One important potential of such models is their predictive ability, which can be significantly beneficial in clinical decision-making. Current TR guidelines provide important direction on when to intervene, but they remain limited in their ability to individualize treatment decisions. The 2025 ESC/EACTS guidelines recommend Heart Team evaluation before intervention, concomitant tricuspid surgery during left-sided valve surgery in patients with severe TR, isolated surgery for symptomatic severe primary TR in the absence of severe RV dysfunction or severe pulmonary hypertension, and consideration of surgery in asymptomatic severe primary TR once RV dilatation or deterioration in RV function develops. They also support consideration of transcatheter treatment in high-risk patients with symptomatic severe TR despite optimal medical therapy, provided severe RV dysfunction and precapillary pulmonary hypertension are absent. Similarly, the 2025 ACC Expert Consensus Decision Pathway proposes a five-step framework—identify, define, assess, treat, and follow-up—and emphasizes multidisciplinary, shared decision-making. Despite these advances, current guidelines still rely largely on threshold-based categories such as “symptomatic severe TR” or “high risk” and do not offer truly individualized prognostic estimates. In particular, they do not predict a given patient’s survival over time, identify when the opportunity for beneficial intervention may be closing, or estimate the relative benefit of surgery, transcatheter therapy, or medical management for that individual. This is where artificial intelligence and machine learning survival models may add value. Methods such as Random Survival Forests and DeepSurv can generate patient-specific survival predictions by integrating multiple clinical variables simultaneously. Random Survival Forests, for example, construct many bootstrap survival trees, use random subsets of predictors at each split, estimate cumulative hazard and survival within terminal nodes, and then aggregate these results into an individualized survival curve. Applied to TR, such models could combine echocardiographic, laboratory, and clinical variables to estimate a patient’s probability of survival at 6 months, 1 year, or 2 years. This level of prognostic precision is more informative than guideline categories alone. These models could be incorporated into clinical practice at the point of Heart Team review. Routinely collected data from echocardiography, laboratory testing, and clinical assessment could be entered into a decision-support tool that provides individualized survival curves alongside guideline-based recommendations. In this setting, AI/ML models could help identify patients earlier within the therapeutic window when intervention is most likely to be beneficial, while also enabling more precise discussions about expected prognosis with surgery, transcatheter therapy, or medical management.

The proposed AI-based model should not be viewed merely as an isolated prognostic tool. Rather, one of its most relevant clinical applications may be as a triage instrument to identify patients at high risk of mortality while they are still candidates for multidisciplinary Heart Team evaluation and timely intervention. This is particularly important because the treatment landscape for severe tricuspid regurgitation (TR) has recently evolved from a predominantly surgical paradigm toward anatomy- and risk-adapted transcatheter interventions, making early identification of high-risk patients increasingly critical before the therapeutic window closes. For patients considered high-risk for surgery, less invasive alternatives have emerged, including transcatheter tricuspid valve interventions such as transcatheter edge-to-edge repair (T-TEER), annuloplasty, orthotopic valve replacement, and heterotopic caval valve implantation. As summarized in [57], these therapies are reshaping the management of severe TR, although optimal patient selection and timing still remain unresolved. In this context, AI-based survival models may help identify patients at risk of clinical deterioration who could benefit from earlier referral to a dedicated Heart Team. Although surgery remains guideline-supported, particularly when performed concomitantly with left-sided valve surgery, isolated tricuspid surgery has historically been associated with substantial perioperative risk, with older national series reporting in-hospital mortality around 9–11%, and more recent STS data suggesting lower operative mortality but persistent composite morbidity/mortality approaching 30% [57]. Contemporary transcatheter options, including T-TEER with TriClip or PASCAL devices, orthotopic transcatheter tricuspid valve replacement with EVOQUE, and heterotopic bicaval implantation with TricValve, can be an alternative in such cases. For instance, T-TEER has demonstrated consistent TR reduction, as well as symptomatic and quality-of-life improvement, across studies such as TRILUMINATE, bRIGHT, Tri.Fr, CLASP TR, and PASTE. However, procedural success remains highly dependent on anatomical and imaging factors, including leaflet grasping feasibility, coaptation gap size, jet location, chordal complexity, and imaging quality [58,59,60,61]. For anatomies less suitable for repair, EVOQUE TTVR may provide more complete elimination of tricuspid regurgitation (TR). In the TRISCEND study, TR was reduced to mild or less in 97.6% of patients, accompanied by marked improvement in NYHA functional class. However, TRISCEND II also highlighted important procedural risks, including bleeding and new pacemaker implantation [49]. For patients who are unsuitable for corrective repair or replacement, TricValve offers a palliative approach by reducing caval backflow and systemic congestion without directly treating the native valve lesion. In the TRICUS and TRICUS EURO studies, 95.5% of high-risk nonsurgical patients experienced improvement in quality of life, functional status, or walking distance [62,63]. These device-specific differences underscore that survival risk should be interpreted alongside procedural feasibility, taking into account both clinical and anatomic risk stratification tools such as TRI-SCORE, TRIVALVE, and GLIDE, as well as multimodality imaging findings, right ventricular function, pulmonary hypertension, renal and hepatic congestion, and atrial fibrillation [47,57]. In this context, the proposed AI-based model may help identify patients earlier in the disease course, before progression to irreversible right ventricular dysfunction, severe pulmonary hypertension, end-organ congestion, or unfavorable anatomy that may preclude repair, replacement, or even palliative therapies.

Table 3 illustrates the results of the multivariable Cox regression model. We can observe that the model has identified several independent predictors of adverse survival. Increasing age and cumulative tobacco exposure (pack-years) were both associated with a modest but statistically significant rise in mortality risk. Each additional year of age increases the hazard by ~4%, reflecting the accumulating burden of comorbidities and reduced cardiac reserve in older patients. Importantly, markers of right heart remodeling—right ventricular (RV) dilation, right ventricular hypertrophy, and right atrial (RA) enlargement also emerged as strong predictors of mortality, underscoring the critical impact of chronic volume overload on right-sided cardiac structure and function. RV enlargement denotes advanced volume overload and maladaptive remodeling under chronic TR, translating into a 63% higher risk of death compared to patients without significant dilation. Furthermore, RV hypertrophy reflects long-standing pressure and volume stress; its strong association with mortality (a nearly two-fold increase). Finally, the presence of non-ischemic myocardial scar conferred a 72% increase in hazard, suggesting that underlying myocardial disease further compromises prognosis in TR. The presence of myocardial fibrosis—even in the absence of coronary artery disease—portends worse outcomes, likely by impairing both systolic and diastolic function of the right heart.

**Figure 4:**
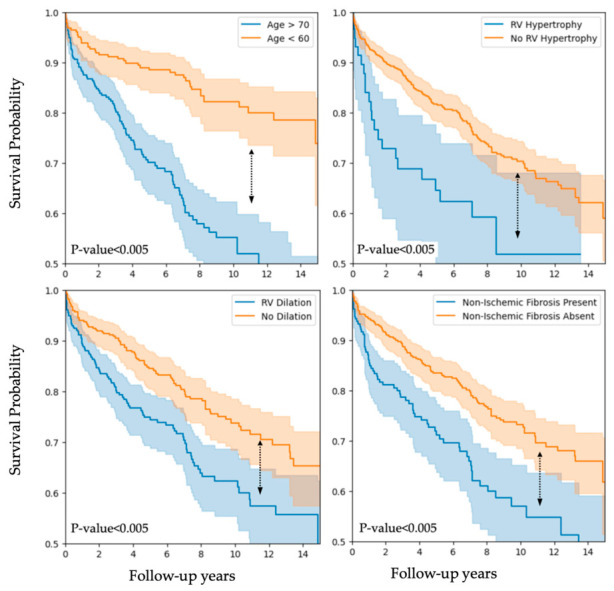
Kaplan–Meier curves for selected clinically relevant features. Arrow indicates the *p*-value for the difference between the graphs.

The significance of these covariates can be further validated by examining the Kaplan–Meier curves for various features—age, RV dilation, Non-Ischemic Scar Presence and RV hypertrophy—as shown in Figure 4. These curves exhibit noticeable divergences, confirming the significance of these covariates. We can see that patients older than 70 years exhibited significantly lower survival compared with those under 60, with an early divergence of the survival curves (Figure 4, top left). This finding aligns with our Cox model analysis (HR 1.04 per year) and likely reflects age-related declines in cardiac reserve, frailty, and comorbidity burden. Clinicians should therefore maintain a high index of suspicion for decompensation in elderly TR patients and consider earlier intervention when clinically appropriate. The survival disadvantage associated with RV dilation (Figure 4, bottom left) highlights its independent association with mortality. Chronic volume overload in TR drives adverse remodeling of the RV, leading to progressive systolic dysfunction. Based on this, imaging evidence of RV enlargement could serve as a red flag for escalating the intensity of medical therapy. Furthermore, patients with non-ischemic fibrosis on cardiac magnetic resonance exhibited lower survival compared to those without fibrosis (Figure 4, bottom right). This observation corroborates the elevated hazard seen in our multivariable model and highlights the additive risk conferred by diffuse myocardial scarring, which may impair both contractile function and ventricular compliance. The most pronounced separation of survival curves was observed for RV hypertrophy (Figure 4, top right), consistent with its highest hazard ratio. Hypertrophic remodeling of the RV in response to chronic pressure and volume stress appears to mark an advanced stage of disease with elevated mortality risk. Early detection of concentric or eccentric hypertrophy—via echocardiography or CMR—could prompt urgent evaluation for corrective interventions before irreversible myocardial changes occur.

These findings are in line with the literature highlighting that patients with moderate-to-severe TR appear to face a mortality risk that is substantially higher than that seen in the general population. In a large meta-analysis of 70 studies including 32,601 patients, moderate/severe TR was associated with nearly double the risk of all-cause mortality compared with no/mild TR (RR 1.95, 95% CI 1.75–2.17), and a graded relationship was seen across severity categories, with mortality risk rising from mild TR (RR 1.25) to moderate TR (RR 1.61) and severe TR (RR 3.44) relative to no TR [64]. This signal is also evident in primary cohort data: Nath et al. reported 1-year survival of 91.7% with no TR, 90.3% with mild TR, 78.9% with moderate TR, and 63.9% with severe TR, showing an early and steep separation in survival curves once TR reaches at least moderate severity [65]. More recently, the TRIO cohort found a 5-year probability of death of 53% for moderate TR, 63% for moderate–severe TR, and 71% for severe TR, underscoring that clinically significant TR is not a benign echocardiographic finding but a marker of sustained excess risk [40]. By contrast, in the overall U.S. population, the 2024 age-adjusted all-cause death rate was 722.1 per 100,000, or about 0.72% per year, while even among older adults the annual death rate was about 1.77% for ages 65–74 and 4.26% for ages 75–84. Thus, the available evidence suggests that moderate-to-severe TR carries a mortality burden far above background all-cause mortality at large. This manuscript was prepared in accordance with the TRIPOD + AI reporting guideline for clinical prediction model studies.

## 5. Conclusions and Future Work

Machine learning-based survival models, particularly models with the ability to model complex nonlinear relationships such as RSF and DeepSurv, can benefit individualized risk stratification in patients with advanced TR. Structural abnormalities of the right heart and myocardial fibrosis were among the most significant predictors of mortality, highlighting the importance of early detection and timely intervention. Integrating AI-driven survival prediction into clinical workflows could potentially benefit decision-making and enable more personalized management of TR. By leveraging predictive AI models, clinicians can better stratify risk, optimize the timing of intervention, and customize follow-up intensity. Future work should focus on using AI to design automated end-to-end systems to extract these covariates from images, and translating AI risk scores to clinical decision support. Our single-center cohort and retrospective design may limit generalizability. Future prospective studies should validate these findings in larger, multicenter populations and explore whether the incorporation of AI-driven risk models can predict optimal timing of TR interventions. Finally, serial imaging to track progression of RV remodeling may yield dynamic prognostic insights beyond the static baseline assessments presented here.

## Figures and Tables

**Table 1:** Statistics of the patient cohort including demographics, prior procedures, medications, and CMR variables.

Demographics		Vitals	
Age	63.7 ± 15.8	Baseline HR	71.8 ± 26.2
Gender (Male)	51.3%	Hematocrit	38.1 ± 6.5
BMI	26.5 ± 6.1	BSA	1.86 ± 0.3
**Comorbidities**		Weight	75.4 ± 20.1
Event (Death)	25.5%	**Imaging Parameters**	
Hypertension	64.3%	LV EDV-indexed	85.8 ± 37.5
Hyperlipidemia	52.2%	LV ESV-indexed	47.8 ± 36.9
Diabetes	23.5%	RV EDV-indexed	104.9 ± 38.5
Chronic Heart Failure	50.6%	RV ESV-indexed	59.6 ± 31.7
Kidney Disease	14.3%	LV SV-indexed	38.0 ± 13.7
Coronary Artery Disease	47.3%	RV SV-indexed	45.3 ± 15.9
Atrial Fibrillation	39.4%	RV hypertrophy	7.7%
Peripheral Vascular Disease	16.5%	LV hypertrophy	33.3%
Smoking	42.5%	RV dilation	44.4%
**Prior Interventions**		LV dilation	25.0%
Valve intervention	16.3%	RV EF	45.3 ± 12.8
Other Cardiac Intervention	37.5%	LV EF	49.6 ± 18.6
**Medication**		LA enlargement	67.2%
Statin	45.9%	RA enlargement	84.8%
Beta-Blocker	57.1%	NIF	27.6%
Entresto	2.9%	NIsF	26.1%
SGLT2i	2.6%	Ischemic scar	26.4%

**Table 2:** Results of model performance.

Model	Concordance Index	Area Under the Curve
Cox PH-Linear	66%	76%
DeepSurv	72%	78%
Random Survival Forests	78%	82%

**Table 3:** Covariate coefficients estimated by the Cox PH model.

Covariate	Hazard Ratio	Confidence Interval 95%	*p*-Value
Age	1.04	1.03–1.05	<0.005
Gender (Male)	0.76	0.56–1.04	0.08
Pack Years	1.01	1.00–1.01	0.03
Hypertension	0.85	0.62–1.16	0.31
Hyperlipidemia	0.93	0.69–1.25	0.61
Atrial Fibrillation	0.95	0.70–1.28	0.72
RV Dilation	1.62	1.18–2.23	<0.005
LV Dilation	1.01	0.65–1.58	0.96
RV Ejection Fraction	1.00	0.99–1.02	0.56
LV Ejection Fraction	0.99	0.98–1.01	0.33
RV Hypertrophy	1.91	1.13–3.21	0.01
RA Enlargement	1.63	1.25–2.13	<0.005
Baseline HR	1.00	0.99–1.01	0.81
Non-Ischemic Scar	1.72	1.23–2.42	<0.005
Ischemic Scar	1.11	0.77–1.60	0.56

## Data Availability

The datasets generated and/or analyzed during this study are subject to restrictions due to ethical and privacy considerations.

## Supplementary Materials

The following supporting information can be downloaded at https://www.mdpi.com/article/10.3390/jcm15103859/s1: Table S1: Percentage of missing values per variable.

## References

[1] Samim D., Praz F., Cochard B., Brugger N., Ruberti A., Bartkowiak J., Corpataux N., Reineke D., Pilgrim T., Windecker S. (2022). Natural history and mid-term prognosis of severe tricuspid regurgitation: A cohort study. Front. Cardiovasc. Med..

[2] Tan T.C., Mullie L., Flynn A.W., Mehrotra P., Shahian D.M., Nunes M.C.P., Picard M.H., Afilalo J. (2023). Association Between Functional Tricuspid Regurgitation and Mortality Following Cardiac Surgery. JACC Adv..

[3] Sagie A., Schwammenthal E., Newell J.B., Harrell L., Joziatis T.B., Weyman A.E., Levine R.A., Palacios I.F. (1994). Significant tricuspid regurgitation is a marker for adverse outcome in patients undergoing percutaneous balloon mitral valvuloplasty. J. Am. Coll. Cardiol..

[4] Muraru D., Clement A., Badano L.P. (2023). Isolated Tricuspid Regurgitation in the Community: Phenotyping the Patients. European Society of Cardiology. https://www.escardio.org/communities/councils/cardiology-practice/education/cardiopractice/isolated-tricuspid-regurgitation-in-the-community-phenotyping-the-patients/.

[5] Wang T.K.M., Unai S., Xu B. (2021). Contemporary review in the multi-modality imaging evaluation and management of tricuspid regurgitation. Cardiovasc. Diagn. Ther..

[6] Cannata F., Stankowski K., Galasso M., Muratori M., Mancini E., Colombo A., Pontone G., De Marco F., Fazzari F., Mangieri A. (2024). Key Imaging Factors for Transcatheter Management of Tricuspid Regurgitation: Device and Patient Selection. J. Clin. Med..

[7] Sung S.H. (2025). The Evolving Landscape of Tricuspid Regurgitation Management. JACC Asia.

[8] Messika-Zeitoun D., Chan V., Labinaz M., Burwash I.G., Dreyfus J. (2024). Intervention for Tricuspid Valve Regurgitation: Timing Is Key, and Earlier Is Better Than Later. Can. J. Cardiol..

[9] Helman S.M., Herrup E.A., Christopher A.B., Al-Zaiti S.S. (2021). The role of machine learning applications in diagnosing and assessing critical and non-critical CHD: A scoping review. Cardiol. Young.

[10] Tandon A., Mohan N., Jensen C., Burkhardt B.E., Gooty V., Castellanos D.A., McKenzie P.L., Zahr R.A., Bhattaru A., Abdulkarim M. (2021). Retraining Convolutional Neural Networks for Specialized Cardiovascular Imaging Tasks: Lessons from Tetralogy of Fallot. Pediatr. Cardiol..

[11] Rehman M.U., Naseem S., Butt A.U.R., Mahmood T., Khan A.R., Khan I., Khan J., Jung Y. (2025). Predicting coronary heart disease with advanced machine learning classifiers for improved cardiovascular risk assessment. Sci. Rep..

[12] Tseng A.S., Noseworthy P.A. (2021). Prediction of Atrial Fibrillation Using Machine Learning: A Review. Front. Physiol..

[13] Saqib M., Perswani P., Muneem A., Mumtaz H., Neha F., Ali S., Tabassum S. (2024). Machine learning in heart failure diagnosis, prediction, and prognosis: Review. Ann. Med. Surg..

[14] Sabouri M., Rajabi A.B., Hajianfar G., Gharibi O., Mohebi M., Avval A.H., Naderi N., Shiri I. (2023). Machine learning based readmission and mortality prediction in heart failure patients. Sci. Rep..

[15] Sayadi M., Varadarajan V., Sadoughi F., Chopannejad S., Langarizadeh M. (2022). A Machine Learning Model for Detection of Coronary Artery Disease Using Noninvasive Clinical Parameters. Life.

[16] Akella A., Akella S. (2021). Machine learning algorithms for predicting coronary artery disease: Efforts toward an open source solution. Future Sci. OA.

[17] Wang J.X., Li C.P., Cui Z., Liang Y., Wang Y.H., Zhou Y., Liu Y., Gao J. (2025). Machine learning algorithms to predict heart failure with preserved ejection fraction among patients with premature myocardial infarction. Front. Cardiovasc. Med..

[18] Liu L., Bi B., Cao L., Gui M., Ju F. (2024). Predictive model and risk analysis for peripheral vascular disease in type 2 diabetes mellitus patients using machine learning and shapley additive explanation. Front. Endocrinol..

[19] Armoundas A.A., Narayan S.M., Arnett D.K., Spector-Bagdady K., Bennett D.A., Celi L.A., Friedman P.A., Gollob M.H., Hall J.L., Kwitek A.E. (2024). Use of artificial intelligence in improving outcomes in heart disease: A scientific statement from the American Heart Association. Circulation.

[20] Vazquez B., Fuentes-Pineda G., Garcia F., Borrayo G., Prohias J. (2021). Risk markers by sex for in-hospital mortality in patients with acute coronary syndrome: A machine learning approach. Inform. Med. Unlocked.

[21] Zweck E., Spieker M., Horn P., Iliadis C., Metze C., Kavsur R., Tiyerili V., Nickenig G., Baldus S., Kelm M. (2021). Machine Learning Identifies Clinical Parameters to Predict Mortality in Patients Undergoing Transcatheter Mitral Valve Repair. JACC Cardiovasc. Interv..

[22] Kang Y., Sohn S.H., Choi J.W., Hwang H.Y., Kim K.H. (2023). Machine-learning-based prediction of survival and mitral regurgitation recurrence in patients undergoing mitral valve repair. Interdiscip. Cardiovasc. Thorac. Surg..

[23] Penso M., Pepi M., Mantegazza V., Cefalu C., Muratori M., Fusini L., Gripari P., Ghulam Ali S., Caiani E.G., Tamborini G. (2021). Machine Learning Prediction Models for Mitral Valve Repairability and Mitral Regurgitation Recurrence in Patients Undergoing Surgical Mitral Valve Repair. Bioengineering.

[24] Zhou N., Zhang K., Qiao B., Chen C., Guo X., Fu W., Zheng J., Du J., Dong R. (2024). Personalized risk prediction of mortality and rehospitalization for heart failure in patients undergoing mitral valve repair surgery. Front. Cardiovasc. Med..

[25] Cipollone P., Pierucci N., Matteucci A., Palombi M., Laviola D., Bruti R., Vinciullo S., Bernardi M., Spadafora L., Cersosimo A. (2025). Artificial Intelligence in Cardiac Electrophysiology: A Comprehensive Review. J. Pers. Med..

[26] Tang S., Razeghi O., Kapoor R., Alhusseini M.I., Fazal M., Rogers A.J., Bort M.R., Clopton P., Wang P.J., Rubin D.L. (2022). Machine Learning-Enabled Multimodal Fusion of Intra-Atrial and Body Surface Signals in Prediction of Atrial Fibrillation Ablation Outcomes. Circ. Arrhythm. Electrophysiol..

[27] Fox S.R., Toomu A., Gu K., Kang J., Sung K., Han F.T., Hoffmayer K.S., Hsu J.C., Raissi F., Feld G.K. (2024). Impact of artificial intelligence arrhythmia mapping on time to first ablation, procedure duration, and fluoroscopy use. J. Cardiovasc. Electrophysiol..

[28] Deisenhofer I., Albenque J.-P., Busch S., Gitenay E., Mountantonakis S.E., Roux A., Horvilleur J., Bakouboula B., Oza S., Abbey S. (2025). Artificial intelligence for individualized treatment of persistent atrial fibrillation: A randomized controlled trial. Nat. Med..

[29] Roguin A., Abu Dogosh A., Feld Y., Konigstein M., Lerman A., Koifman E. (2021). Early Feasibility of Automated Artificial Intelligence Angiography Based Fractional Flow Reserve Estimation. Am. J. Cardiol..

[30] Cox D.R. (1972). Regression models and life-tables. J. R. Stat. Soc. Ser. B (Methodol.).

[31] Ishwaran H., Kogalur U.B., Blackstone E.H., Lauer M.S. (2008). Random survival forests. Ann. Appl. Stat..

[32] Katzman J.L., Shaham U., Cloninger A., Bates J., Jiang T., Kluger Y. (2018). DeepSurv: Personalized treatment recommender system using a Cox proportional hazards deep neural network. BMC Med. Res. Methodol..

[33] Villar-Calle P., Pai V., Zhang R.S., Reza M., Jin L., Axman R., Falk Z., Falco G., RoyChoudhury A., Chen S. (2025). Nonischemic Septal Fibrosis in Functional Tricuspid Regurgitation Provides Incremental Stratification of Adverse Remodeling and Prognosis. JACC Cardiovasc. Imaging.

[34] Almani M.U., Khan R., Fatima N., Yousuf M., Amanullah A. (2025). Impact of Tricuspid Regurgitation on the Clinical Outcomes of Patients with Heart Failure. Public Health Chall..

[35] Singh S., Mohammed A.S., DeJonge J., Puskoor A.V., Desai R., Ghani M.U., Ghantasala P., Fattal P.G., Sareen N. (2026). Impact of moderate to severe tricuspid regurgitation on long-term clinical outcomes in heart failure: A systematic review and meta-analysis of 456,353 patients. Heart Lung.

[36] Kelly B.J., Luxford J.M.H., Butler C.G., Huang C.-C., Wilusz K., Ejiofor J.I., Rawn J.D., Fox J.A., Shernan S.K., Muehlschlegel J.D. (2018). Severity of tricuspid regurgitation is associated with long-term mortality. J. Thorac. Cardiovasc. Surg..

[37] Messika-Zeitoun D., Verta P., Gregson J., Pocock S.J., Boero I., Feldman T.E., Abraham W.T., Lindenfeld J., Bax J., Leon M. (2020). Impact of tricuspid regurgitation on survival in patients with heart failure: A large electronic health record patient-level database analysis. Eur. J. Heart Fail..

[38] Benfari G., Antoine C., Miller W.L., Thapa P., Topilsky Y., Rossi A., Michelena H.I., Pislaru S., Enriquez-Sarano M. (2019). Excess Mortality Associated With Functional Tricuspid Regurgitation Complicating Heart Failure With Reduced Ejection Fraction. Circulation.

[39] Fender E.A., Petrescu I., Ionescu F., Zack C.J., Pislaru S.V., Nkomo V.T., Cochuyt J.J., Hodge D.O., Nishimura R.A. (2019). Prognostic Importance and Predictors of Survival in Isolated Tricuspid Regurgitation: A Growing Problem. Mayo Clin. Proc..

[40] Lara-Breitinger K.M., Scott C.G., Nkomo V.T., Pellikka P.A., Kane G.C., Chaliki H.P., Shapiro B.P., Eleid M.F., Alkhouli M., Greason K.L. (2022). Tricuspid Regurgitation Impact on Outcomes (TRIO): A Simple Clinical Risk Score. Mayo Clin. Proc..

[41] Long A., Finer J., Hartman H., Hartzel D., Jing L., Kelsey C., Rocha D., Ruhl J., Vanmaanen D., Elnabawi Y. (2025). Deep learning for echocardiographic assessment and risk stratification of aortic, mitral, and tricuspid regurgitation: The DELINEATE-regurgitation study. Eur. Heart J..

[42] Vrudhula A., Vukadinovic M., Haeffele C., Kwan A.C., Berman D., Liang D., Siegel R., Cheng S., Ouyang D. (2025). Automated Deep Learning Phenotyping of Tricuspid Regurgitation in Echocardiography. JAMA Cardiol..

[43] Diao X., Xu W., Cheng H., Zhou Y., Liu Y., Huo Y., Lu J., Huang J., He J., Liu F. (2025). SPEED-TR: A self-distilled and pre-trained transformer model for enhanced ECG detection of tricuspid regurgitation. npj Digit. Med..

[44] Xie S., Liu H., Su L., Shen J., Miao J., Huang D., Zhou M., Liu H., Li Y., Yin L. (2024). A deep learning-based method for assessing tricuspid regurgitation using continuous wave Doppler spectra. Sci. Rep..

[45] Yang T., Wang Y., Zhu G., Liu W., Cao J., Liu Y., Lu F., Yang J. (2025). TRI-PLAN: A deep learning-based automated assessment framework for right heart assessment in transcatheter tricuspid valve replacement planning. Int. J. Cardiol..

[46] Aoyama G., Zhou Z., Zhao L., Zhao S., Kawashima K., Chapman J.V., Asami M., Nozaki Y., Fujimoto S., Sakaguchi T. (2024). Automatic tricuspid valve annulus extraction and measurement from computed tomography images. Inform. Med. Unlocked.

[47] Bartkowiak J., Vivekanantham H., Kassar M., Dernektsi C., Agarwal V., Lebehn M., Windecker S., Brugger N., Hahn R.T., Praz F. (2024). Computed tomography anatomic predictors of outcomes in patients undergoing tricuspid transcatheter edge-to-edge repair. J. Cardiovasc. Comput. Tomogr..

[48] Shimoda T.M., Ueyama H.A., Miyamoto Y., Watanabe A., Gotanda H., Kolte D., Latib A., Kaneko T., Zajarias A., Elmariah S. (2025). Comparison of Transcatheter Versus Surgical Tricuspid Repair Among Patients With Tricuspid Regurgitation: Two-Year Results. Circ. Cardiovasc. Interv..

[49] Hahn R.T., Makkar R., Thourani V.H., Makar M., Sharma R.P., Haeffele C., Davidson C.J., Narang A., O’neill B., Lee J. (2025). Transcatheter Valve Replacement in Severe Tricuspid Regurgitation. N. Engl. J. Med..

[50] Ancona F., Bellettini M., Polizzi G., Paci G., Margonato D., Ingallina G., Stella S., Fiore G., Tavernese A., Belli M. (2024). Short-term outcome after isolated tricuspid valve surgery: Prognostic role of right ventricular strain. Eur. J. Cardiothorac. Surg..

[51] Badano L.P., Penso M., Tomaselli M., Kim K., Clement A., Radu N., Hong G.-R., Hădăreanu D.R., Buta A., Delcea C. (2025). Advanced echocardiography and cluster analysis to identify secondary tricuspid regurgitation phenogroups at different risk. Rev. Esp. Cardiol. (Engl. Ed.).

[52] Heitzinger G., Spinka G., Koschatko S., Baumgartner C., Dannenberg V., Halavina K., Mascherbauer K., Nitsche C., Dona C., Koschutnik M. (2023). A streamlined, machine learning-derived approach to risk-stratification in heart failure patients with secondary tricuspid regurgitation. Eur. Heart J. Cardiovasc. Imaging.

[53] Stocker T.J., Hertell H., Orban M., Braun D., Rommel K.-P., Ruf T., Ong G., Nabauer M., Deseive S., Fam N. (2021). Cardiopulmonary Hemodynamic Profile Predicts Mortality After Transcatheter Tricuspid Valve Repair in Chronic Heart Failure. JACC Cardiovasc. Interv..

[54] Fortmeier V., Lachmann M., Stolz L., von Stein J., Rommel K.-P., Kassar M., Gerçek M., Schöber A.R., Stocker T.J., Omran H. (2025). Simplified Outcome Prediction in Patients Undergoing Transcatheter Tricuspid Valve Intervention by Survival Tree-Based Modelling. JACC Adv..

[55] Brener M.I., Lurz P., Hausleiter J., Rodés-Cabau J., Fam N., Kodali S.K., Rommel K.-P., Muntané-Carol G., Gavazzoni M., Nazif T.M. (2022). Right Ventricular-Pulmonary Arterial Coupling and Afterload Reserve in Patients Undergoing Transcatheter Tricuspid Valve Repair. J. Am. Coll. Cardiol..

[56] Deb B., Scott C., Pislaru S.V., Nkomo V.T., Kane G.C., Alkhouli M., A Crestanello J., Arruda-Olson A., A Pellikka P., Anand V. (2023). Machine learning facilitates the prediction of long-term mortality in patients with tricuspid regurgitation. Open Heart.

[57] Sanfilippo C., Frazzetto M., Bonanni M., Matteucci A., Leo L.A., Umar R., Imperatore G., Castro P.d.C., Attizzani G., Sangiorgi G.M. (2025). Transcatheter treatment of tricuspid regurgitation: A state of art review. Cardiovasc. Revasc. Med..

[58] Huynh K. (2023). Transcatheter repair for severe tricuspid regurgitation. Nat. Rev. Cardiol..

[59] Kar S., Makkar R.R., Whisenant B.K., Hamid N., Naik H., Tadros P., Price M.J., Singh G., Schwartz J.G., Kapadia S. (2025). Two-Year Outcomes of Transcatheter Edge-to-Edge Repair for Severe Tricuspid Regurgitation: The TRILUMINATE Pivotal Randomized Controlled Trial. Circulation.

[60] Kodali S., Hahn R.T., Makkar R., Makar M., Davidson C.J., Puthumana J.J., Zahr F., Chadderdon S., Fam N., Ong G. (2023). Transfemoral tricuspid valve replacement and one-year outcomes: The TRISCEND study. Eur. Heart J..

[61] Kodali S.K., Hahn R.T., Davidson C.J., Narang A., Greenbaum A., Gleason P., Kapadia S., Miyasaka R., Zahr F., Chadderdon S. (2023). 1-Year Outcomes of Transcatheter Tricuspid Valve Repair. J. Am. Coll. Cardiol..

[62] Blasco-Turrión S., Briedis K., Estévez-Loureiro R., Sánchez-Recalde A., Cruz-González I., Pascual I., Mascherbauer J., Abdul-Jawad Altisent O., Nombela-Franco L., Pan M. (2024). Bicaval TricValve Implantation in Patients With Severe Symptomatic Tricuspid Regurgitation: 1-Year Follow-Up Outcomes. JACC Cardiovasc. Interv..

[63] Donal E., Dreyfus J., Leurent G., Coisne A., Leroux P.-Y., Ganivet A., Sportouch C., Lavie-Badie Y., Guerin P., Rouleau F. (2025). Transcatheter Edge-to-Edge Repair for Severe Isolated Tricuspid Regurgitation: The Tri.Fr Randomized Clinical Trial. JAMA.

[64] Wang N., Fulcher J., Abeysuriya N., McGrady M., Wilcox I., Celermajer D., Lal S. (2019). Tricuspid regurgitation is associated with increased mortality independent of pulmonary pressures and right heart failure: A systematic review and meta-analysis. Eur. Heart J..

[65] Nath J., Foster E., Heidenreich P.A. (2004). Impact of tricuspid regurgitation on long-term survival. J. Am. Coll. Cardiol..

