# Supplementary material for: Prognostic Modeling of Tricuspid Valve Regurgitation Outcomes Using Machine Learning-Based Survival Analysis
Source: J Clin Med. 2026 May 17;15(10):3859. doi: 10.3390/jcm15103859 (PMC13207346; doi:10.3390/jcm15103859)
Supplement: Supplementary file 1 [file jcm-15-03859-s001.zip › jcm-4299060-supplementary.pdf]

Supplementary material

Table S1. Percentage of missing values per variable

Variable	Missing (%)	Variable	Missing (%)
**Gender**	0.00 %	**RVEF**	0.42 %
**Age**	0.00 %	**Non-Ischemic Scar**	0.11 %
**Hypertenstion**	0.53 %	**Ischemic Scar**	0.11 %
**Hyperlipidemia**	0.84 %	**HR Baseline**	59.0 %
**Atrial Fibrilation**	0.95 %	**RV Hypertrophy**	0.00 %
**Smoking Years**	1.40 %	**RA Enlargement**	18.65 %
**LV Dilation**	0.11 %	**RV Dilation**	0.00 %
**LVEF**	0.11 %		
